# Midkine Serum Levels in Inflammatory and Non-Inflammatory Dilated Cardiomyopathy

**DOI:** 10.3390/biomedicines13020504

**Published:** 2025-02-18

**Authors:** Ulrich Grabmaier, Bartolo Ferraro, Kristin Lehnert, Astrid Petersmann, Stephan B. Felix, Ludwig T. Weckbach

**Affiliations:** 1 Medizinische Klinik und Poliklinik I, Ludwig-Maximilians-University Hospital, 81377 Munich, Germany; ulrich.grabmaier@med.uni-muenchen.de (U.G.); bartolo.ferraro@med.uni-muenchen.de (B.F.); 2 Deutsches Zentrum für Herz-Kreislauf-Forschung (DZHK) (German Centre for Cardiovascular Research), Partner Site Munich, 80333 Munich, Germany; 3 Institute of Cardiovascular Physiology and Pathophysiology, Biomedical Center, Ludwig-Maximilians-University, 82152 Planegg-Martinsried, Germany; 4 Department of Internal Medicine B, University Medicine Greifswald, 17475 Greifswald, Germany; kristin.lehnert@uni-greifswald.de (K.L.); felix@uni-greifswald.de (S.B.F.); 5 Deutsches Zentrum für Herz-Kreislauf-Forschung (DZHK) (German Centre for Cardiovascular Research), Partner Site Greifswald, 17475 Greifswald, Germany; 6 Institute of Clinical Chemistry and Laboratory Medicine, University Medicine Greifswald, 17475 Greifswald, Germany; petersmann.astrid@klinikum-oldenburg.de; 7 Institute of Clinical Chemistry and Laboratory Medicine, University Medicine Oldenburg, 26133 Oldenburg, Germany; 8 Walter Brendel Centre of Experimental Medicine, Ludwig-Maximilians-University Hospital, 81377 Munich, Germany

**Keywords:** dilated cardiomyopathy, midkine, biomarker, heart failure

## Abstract

**Objectives**: This retrospective study examines midkine, an inflammatory cytokine, as a potential serological biomarker to distinguish dilated cardiomyopathy (DCM) and inflammatory dilated cardiomyopathy (DCMi). Identifying such a biomarker is crucial for effective treatment of these two entities. **Methods**: The study included 54 patients with heart failure, reduced left ventricular systolic function, and suspected cardiac inflammation. Endomyocardial biopsies were obtained from all 54 patients to differentiate between DCM and DCMi. Blood sera were collected from these patients the same day the endomyocardial biopsy was performed and compared with those of 13 age-matched healthy individuals for different measurements such as midkine and NT-proBNP. Patients were followed up to a median of 194 days after the baseline visit. **Results**: Endomyocardial biopsies from patients with DCMi were associated with more infiltrating immune cells such as CD68^+^ macrophages and CD3^+^ T cells and a more frequent presence of a viral genome than those from patients with DCM. Both groups showed similar improvements in LV function and dimensions over time. MK serum levels were significantly higher in DCM/ DCMi patients than in healthy individuals but did not differ significantly between DCM and DCMi. MK levels did not significantly correlate with NYHA class, NT-proBNP, LVEDD, or LVEF, except for a weak correlation with LVEF at follow-up. **Conclusions**: Midkine serum levels were significantly higher in patients with a DCM phenotype and severely reduced systolic function. However, these levels could not distinguish between DCM and DCMi and showed no correlation with baseline or follow-up parameters. Therefore, midkine cannot be used as a biomarker to distinguish between DCM and DCMi.

## 1. Introduction

Heart failure represents a major clinical and epidemiological syndrome with increasing prevalence and high morbidity and mortality. Brain natriuretic peptide (BNP) or the N-terminal BNP (NT-proBNP) are routinely used as biomarkers in clinical practice [1]. As inflammation is a critical feature of heart failure, different inflammatory markers including C-reactive protein (CRP) or the interleukins (IL) 1, 6, and 18 represent interesting candidates as additional biomarkers. Furthermore, the pro-inflammatory cytokine midkine (MK) has been investigated as a biomarker in heart failure [2]. Midkine is a 13-kDa heparin-binding cytokine and growth factor with a relevant role in various physiological and pathophysiological processes due to its pro-inflammatory, pro-angiogenic, anti-apoptotic, and anti-infective functions [3]. Early studies by Kadomatsu et al. [4] showed that MK is up-regulated during mid-gestation in mice embryos, but its expression in adult mice is confined to the kidney. Numerous cell types such as monocytes, macrophages, monocyte-derived dendritic cells (mDCs), and endothelial cells have been identified to produce MDK under basal and pathological conditions [5]. It has been shown that MK plays a role in vascular remodeling, angiogenesis, and inflammation, in conditions such as atherosclerosis, myocardial infarction, and hypertension [3]. Moreover, MK levels were found to be elevated in the serum of patients with sepsis and septic shock when compared to control subjects [6]. In the context of dilated cardiomyopathy (DCM), a study by Danielle Jeffrey indicated that midkine is up-regulated in both the plasma and cardiac tissue of pediatric patients with DCM. Additionally, the presence of circulating midkine contributes to pathological changes in gene expression by increasing the expression of the atrial natriuretic factor (ANF) and b-type natriuretic peptide (BNP) [7]. Overall, midkine shows potential as a biomarker in various pathological conditions, including applications for early detection, disease progression monitoring, and treatment response assessment. Our research group has shown that MK was critically involved in cardiac inflammation in a mouse model of experimental autoimmune myocarditis. Blocking MK reduced leukocyte infiltration and ameliorated subsequent fibrosis and heart failure [8]. Although myocarditis is commonly self-limiting, some patients will develop progressive heart failure due to ongoing cardiac inflammation, which is referred to as inflammatory dilated cardiomyopathy (DCMi) [9,10]. As the diagnosis of DCMi may influence therapy (e.g., immunomodulation), we examined whether MK serum levels may differentiate DCMi from non-inflammatory DCM.

## 2. Material and Methods

### 2.1. Patient Study Design

Fifty-four patients (52 male, 2 female) with heart failure with reduced left ventricular systolic function and suspected cardiac inflammation gave written informed consent to be prospectively enrolled into a cardiomyopathy registry (2004–2013, University Hospital of Greifswald). Blood sera were collected and compared with those of 13 male, age-matched, healthy individuals (mean age 53.8 ± 2.8) that were used as the control. Relevant coronary artery disease as a pathophysiological cause for heart failure was excluded in all patients using coronary angiography. Endomyocardial biopsies were obtained from all 54 patients to differentiate between DCM and DCMi. DCMi was defined by the presence of >14 inflammatory cells/mm^2^, in addition to the enhanced expression of human leukocyte antigen (HLA) class II molecules as previously described [9]. Pregnant patients, patients with known autoimmune disease or treated with anti-inflammatory medication, and patients <18 years of age were excluded, as well as patients who received therapeutic dosages of heparin, as administration of such dosages can dramatically increase MK serum levels [11]. As MK levels are also influenced by renal failure, only patients with a glomerular filtration rate (eGFR) above 60 mL/min were included in the study. Blood samples were collected on the same day the endomyocardial biopsy was performed. Patients were followed up to a median of 194 [IQR 156–265] days after the baseline visit. The investigation conforms with the principles outlined in the Declaration of Helsinki.

### 2.2. ELISA

Midkine serum levels were measured using a colorimetric enzyme-linked immunosorbent assay (Midkine ELISA, CELLMID, Sydney, Australia). To assess the levels of circulating N-terminal pro-B-type natriuretic peptide (NT-proBNP), we used the enzyme-linked immunosorbent assay (ELISA; Biomedica, Vienna, Austria).

### 2.3. Glomerular Filtration Rate (GFR)

Creatinine was measured in plasma according to routine diagnostic methods and GFR was calculated with the MDRD formula.

### 2.4. Immunohistochemical Analysis

The data collected from the endomyocardial biopsies were obtained from hospital records. For this analysis, we relied on the expertise of the individuals conducting the work. The pathologists from the University Hospital Tübingen were part of the German Research Foundation (DFG), Transregional Collaborative Research Centre “Inflammatory Cardiomyopathy—Molecular Pathogenesis and Therapy” project that enabled the cardiomyopathy registry that is the source of samples and data for this project. The immunohistochemistry protocol used has been published in other analyses from the registry [9,12]. For the immunohistochemical analyses, we examined cardiac inflammation in paraffin-embedded tissue sections using the avidin-biotin-immunoperoxidase method, adhering to the manufacturer’s protocol (Vectastatin Elite ABC kit, Vectastatin^®^, Vector Laboratories, Newark, CA, USA) [9,12]. We employed monoclonal antibodies to assess cardiac cell infiltration, specifically evaluating CD3^+^ T-lymphocytes (Novocastra Laboratories, Newcastle upon Tyne, UK) and CD68^+^ macrophages (DAKO, Hamburg, Germany), in accordance with the guidelines for the Definition and Classification of Cardiomyopathies that were in place at the time of data collection [13]. To evaluate HLA class II expression, we utilized an anti-HLA-DR-α antibody (DAKO, Hamburg, Germany) [9].

### 2.5. Cardiac Parameter

Echocardiographic evaluations were conducted on all patients using 2-dimensional echocardiography during their initial hospital admission, following the American College of Cardiology/American Heart Association guidelines applicable at the time of data collection [14]. The left ventricular ejection fraction was measured using the biplane Simpson method, while left ventricular dilation was assessed by measuring the left ventricular end-diastolic diameter (LVEDD).

### 2.6. Viral Load EMB

For the detection of viral genomes in myocardial biopsies, a nested PCR/RT-PCR was performed as described previously [15].

### 2.7. Statistical Analysis

All data are presented as the median with 95% confidence interval (CI). Comparisons of dichotomous variables were analyzed by the Chi-Square-test (χ^2^). The Kolmogorov–Smirnov test was performed to analyze the normal distribution of continuous variables. Not normally distributed data were evaluated using a two-tailed Mann–Whitney U-test. Normally distributed variables were evaluated by a two-sided *t*-test. Correlation was tested using Pearson’s R. A *p*-value of <0.05 was considered statistically significant. For the statistical analysis, SPSS statistics (version 26, IBM, Armonk, NY, USA) was used.

## 3. Results

The clinical characteristics of patients with DCM and DCMi are depicted in Table 1. Twenty-nine patients had DCM, of whom three had a presumed alcohol toxic etiology, one had a possible familial/genetic DCM, and one had a presumed decompensated hypertensive heart disease. Twenty-five patients showed immunohistochemical evidence of inflammatory DCM. To adjust for possible confounders, groups were matched for NYHA functional class, NT-proBNP levels, renal function, and left ventricular ejection fraction (LVEF), as well as the left ventricular end-diastolic diameter (LVEDD). Therefore, these parameters were similar in both groups. Major histocompatibility complex-II (MHC-II) upregulation, the number of infiltrated CD68^+^ macrophages, as well as CD3^+^ T cells in the endomyocardial biopsy were significantly higher in the DCMi compared to the DCM group, as these parameters were used to differentiate between both entities. Further, detection of a viral genome was more frequent in DCMi patients. Follow-up characteristics are shown in Table 2.

**Figure 1:**
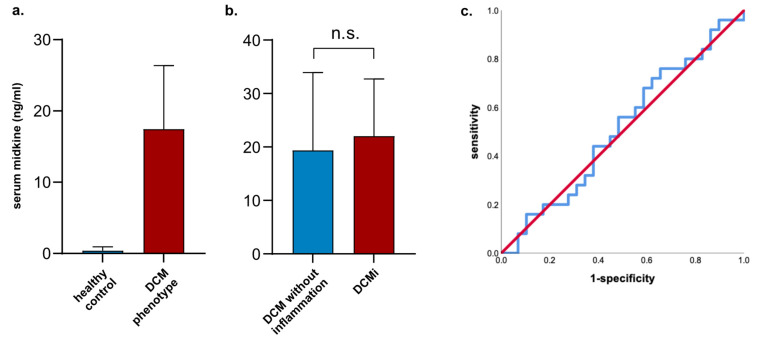
Evaluation of MK serum levels for diagnosis of DCMi. (**a**) Compared to age-matched healthy controls, MK serum levels in patients with heart failure and a DCM phenotype were markedly increased (median 0.4 ng/mL vs. 21.4 ng/mL); *n* = 13 for healthy controls, *n* = 54 for DCM phenotype. (**b**) This shows MK serum levels of patients with DCM without inflammation and DCMi (median 19.4 ng/mL vs. 22.0 ng/mL). (**c**) This shows ROC curve for MK serum levels between both entities. AUC, area under the curve; *n* = 29 for DCM, *n* = 25 for DCMi; n.s., not significant.

In total, nine patients died during follow-up: four patients in the DCM group and five patients in the DCMi group, respectively (13.8% vs. 20% in the DCMi group; *p* = 0.542, Table 2). Both groups showed comparable improvement of LV function and diameters at follow-up 1 (at a median of 95 (IQR 69–108) days after baseline visit, Table 2), and further at follow-up 2 (at a median of 194 (IQR 156–265) after baseline visit, Table 2). In comparison to healthy individuals, MK serum levels were significantly increased in patients with a DCM phenotype (median 0.4 (IQR 0.3–0.7) ng/mL in healthy individuals vs. 21.4 (IQR 5.3–35.0) ng/mL in patients with a DCM phenotype, *p* < 0.001, Figure 1a).

Further, we could not detect any differences in MK serum levels between patients who initially presented with dilated cardiomyopathy in echocardiography and underwent endomyocardial biopsy for further immunohistological differentiation into both entities (median 19.4 (IQR 4.9–38.8) ng/mL in DCM vs. 22.0 (IQR 7.1–34.2) ng/mL in patients with DCMi, *p* = 0.883, Figure 1b). Figure 1c shows the receiver operating characteristic (ROC) curve for MK serum levels, reflecting no separation between DCM and DCMi (area under curve (AUC) of 0.512, 95% CI 0.356–0.668).

To gain further insights into the prognostic value of MK as a biomarker in heart failure, we investigated whether MK serum levels would correlate with functional parameters in heart failure including NYHA functional class, NT-proBNP levels, LVEF, and LVEDD at baseline and follow-up. However, none of these parameters correlated significantly with MK serum levels, except LVEF at follow-up 2 (Pearson’s R 0.348, *p* = 0.047, Table 3).

## 4. Discussion

DCMi reflects a relevant sub-entity of the phenotype DCM. To date, the diagnosis of DCMi relies on the immunohistochemical evaluation of endomyocardial biopsies. A definitive diagnosis is often not established in clinical practice due to safety concerns. Since evidence suggests that cardiac function cannot be improved in patients with DCMi despite receiving optimal drug therapy, left ventricular ejection fraction (LVEF) does improve with immunosuppressive therapy in patients with biopsy-proven virus-negative inflammatory cardiomyopathy [9,16,17]. Therefore, identifying a biomarker to differentiate between the two entities is of critical therapeutic importance. We have recently shown that MK mRNA expression was substantially elevated in the inflamed cardiac tissue in a myocarditis mouse model compared to the control mice [8]. Therefore, we have attempted to identify the inflammatory cytokine MK as a possible serological biomarker to distinguish the DCM entities within the present retrospective study.

MK serum levels were markedly elevated in patients with DCM or DCMi compared to healthy individuals, but did not differ between the two biopsy-distinguished groups. Further, they did not show a correlation with heart failure-classifying parameters.

As we showed in the present study, MK was not differentially concentrated between both groups. This may be explained by the fact that heart failure per se is a highly inflammatory condition, which is accompanied by a significant up-regulation and subsequent release of MK into the circulation. The up-regulation of MK in heart failure has been demonstrated in the failing hearts of pediatric patients with DCM compared to the non-failing control hearts at the RNA level [18]. These findings suggest that up-regulation of MK may be a typical mechanism for failing hearts. However, the source of MK in failing hearts and in the serum of heart failure patients is still undetermined.

Our finding of elevated MK serum levels in DCM patients is in accordance with a study conducted by Kitahara and colleagues [2]. In contrast to our study, Kitahara et al. only observed modestly increased MK serum levels (2–4-fold) in heart failure patients compared to healthy controls. In our study, MK serum levels were approximately 50-fold elevated in patients with a DCM phenotype compared to healthy individuals. This may be due to the fact that the systolic function in our study was substantially reduced (mean 29.5%) and left ventricles were markedly dilated (mean 67.8 mm), whereas systolic function and LVEDD were only mildly abnormal in the study conducted by Kitahara et al. indicating that in our study, heart failure was more progressed [2]. Nevertheless, the serum levels of healthy individuals were comparable to values measured by different working groups, underlying the validity of our data [19,20,21,22,23].

We set out to investigate whether MK serum levels would correlate with baseline and follow-up parameters classifying heart failure in patients. However, we did not observe any correlation between MK serum levels and NYHA functional class, natriuretic peptides, LVEF, or LVEDD at baseline. Only LVEF at follow-up 2 showed a weak correlation, which—in synopsis with the missing correlation with baseline and other follow-up parameters—can be regarded as an outlier. In contrast to our study, Kitahara et al. found a correlation between NYHA functional class and MK serum levels despite only moderately elevated MK serum levels in heart failure patients in that study, which may be due to the larger sample size and a more heterogeneous patient population [2]. In this regard, we would like to emphasize that our study has limitations related to the number of patients and to the gender disproportion, which is predominantly due to the higher incidence and severity of most cardiovascular diseases in males [24].

## 5. Conclusions

In summary, we observed markedly elevated MK serum levels in patients with a DCM phenotype with severely reduced systolic function. However, MK serum levels could not discriminate between DCM and DCMi and showed no correlation with baseline or follow-up parameters. We conclude that midkine cannot be used as a biomarker to distinguish between DCM and DCMi.

## Figures and Tables

**Table 1:** Patients’ characteristics.

Characteristics	All Patients (*n* = 54)	DCM (*n* = 29)	DCMi (*n* = 25)	*p*-Value
Age, mean ± SD	54.0 ± 8.8	55.0 ± 8.1	52.9 ± 9.5	0.404
Duration of symptoms in days, median [IQR]	54 [28–130]	55 [29–122]	51 [23–148]	0.910
Male sex, *n* (%)	52 (96.3)	28 (96.6)	24 (96.0)	0.915
Glomerular filtration rate, mean ± SD, mL/min	80.8 ± 12.4	80.5 ± 10.6	81.1 ± 14.4	0.849
NYHA functional class, *n*				0.971
I	2	1	1	
II	30	17	13	
III	18	9	9	
IV	4	2	2	
NT-proBNP in pg/mL, median [IQR]; (*n*)	1706 [1075–3868] (30)	1594 [1139–2991] (15)	2032 [900–3973] (15)	0.604
Echo parameters, mean ± SD;				
LVEF, %	29.5 ± 5.7	29.6 ± 5.8	29.4 ± 5.8	0.904
LVEDD, mm	67.8 ± 5.5	67.4 ± 5.1	68.3 ± 5.9	0.578
Immunohistological parameters				
MHC-II upregulation, *n*	23	0	23	<0.001
CD68^+^ macrophages per mm^2^, median [IQR]	13 [10–22]	11 [9–12]	22 [19–26]	<0.001
CD3^+^ T cells per mm^2^, median [IQR]	1 [0–4]	0 [0–2]	4 [2–7]	<0.001
Evidence of viral genome (PCR of EMB), *n* (%)	17 (31.5)	2 (6.9)	15 (60)	<0.001
Patients with >1 virus, *n*	3	1	2	
Adenovirus, *n*	1	0	1	
EBV, *n*	2	1	1	
HHV-6, *n*	8	1	7	
Parvovirus B19, *n*	10	1	9	

CD68 and CD3, cluster of differentiation 68 and 3; DCM, patients with the phenotype of dilated cardiomyopathy without immunohistological evidence of persistent inflammation; DCMi, patients with DCM and immunohistological evidence of inflammation; EBV, Epstein–Barr virus; EMB, endomyocardial biopsy; HHV-6, human herpesvirus 6; LVEF, left ventricular ejection fraction; LVEDD, left ventricular end-diastolic diameter; MHC-II, major histocompatibility complex class 2; NYHA, New York Heart Association functional class; PCR, polymerase chain reaction.

**Table 2:** Follow-up characteristics.

Survival	All Patients (*n* = 54)	DCM (*n* = 29)	DCMi (*n* = 25)	*p*-Value
Death, *n* (%)	9 (16.7)	4 (13.8)	5 (20)	0.542
Echo parameters at follow-up 1 ^#^	All Patients (*n* = 40–44)	DCM (*n* = 19–23)	DCMi (*n* = 21)	*p*-value
LVEF in %, mean ± SD	38.5 ± 8.6	38.0 ± 9.0	39.0 ± 8.2	0.717
ΔLVEF in %, mean ± SD	9.3 ± 8.5	8.9 ± 8.6	9.7 ± 8.6	0.773
LVEDD in mm, mean ± SD	64.8 ± 7.1	64.0 ± 7.0	65.5 ± 7.3	0.504
ΔLVEDD in mm, median [IQR]	−2.0 [−6.0–1.0]	−2.0 [−7.0–1.0]	−2.0 [−2.0–0.0]	0.587
Echo parameters at follow-up 2 *	All Patients (*n* = 33–34)	DCM (*n* = 17–18)	DCMi (*n* = 16)	*p*-value
LVEF in %, mean ± SD	43.1 ± 8.6	43.2 ± 9.4	43.1 ± 8.0	0.970
ΔLVEF in %, mean ± SD	13.5 ± 8.6	13.2 ± 7.6	13.8 ± 9.8	0.866
LVEDD in mm, mean ± SD	62.8 ± 8.3	63.3 ± 5.9	62.2 ± 10.5	0.693
ΔLVEDD in mm, median [IQR]	−3.5 [−7.3–(−0.5)]	−3.0 [−6.0–(−1.3)]	−4.6 [−13.3–0.95]	0.275

DCM, patients with the phenotype of dilated cardiomyopathy without immunohistological evidence of persistent inflammation; DCMi, patients with DCM and immunohistological evidence of inflammation; LVEF, left ventricular ejection fraction; LVEDD, left ventricular end-diastolic diameter; ΔLVEF, difference between LVEF at follow-up and LVEF at baseline; ΔLVEDD, difference between LVEDD at follow-up and LVEDD at baseline; ^#^ Follow-up 1 was a median of 95 [69–108] after baseline visit; * Follow-up 2 was a median of 194 [156–265] after baseline visit.

**Table 3:** Correlation of serum midkine levels with baseline and follow-up parameters.

Baseline Parameters	Pearson’s R	*p*-Value
NYHA functional class	−0.230	0.094
NT-proBNP in pg/mL	−0.064	0.735
LVEF	0.018	0.899
LVEDD	0.075	0.588
Viral genome detection	−0.005	0.971
Follow-up parameters	Pearson’s R	*p*-value
LVEF at follow-up 1 ^#^, *n* = 44	−0.023	0.883
ΔLVEF at follow-up 1 ^#^, *n* = 44	−0.031	0.844
LVEDD at follow-up 1 ^#^, *n* = 40	−0.273	0.088
ΔLVEDD at follow-up 1 ^#^, *n* = 40	−0.150	0.355
LVEF at follow-up 2 *, *n* = 33	0.348	0.047
ΔLVEF at follow-up 2 *, *n* = 33	0.199	0.268
LVEDD at follow-up 2 *, *n* = 34	−0.103	0.568
ΔLVEDD at follow-up 2 *, *n* = 34	−0.020	0.909

NYHA, New York Heart Association functional class; LVEF, left ventricular ejection fraction; LVEDD, left ventricular end-diastolic diameter; ΔLVEF, difference between LVEF at follow-up and LVEF at baseline; ΔLVEDD, difference between LVEDD at follow-up and LVEDD at baseline; ^#^ Follow-up 1 was a median of 95 [69–108] after baseline visit; * Follow-up 2 was a median of 194 [156–265] after baseline visit.

## Data Availability

The original contributions presented in the study are included in the article. Data collected for the study will not be available online. Further inquiries can be directed to the corresponding author, who will make additional information available upon reasonable request.
